# 1-Octanol emitted by *Oecophylla smaragdina* weaver ants repels and deters oviposition in Queensland fruit fly

**DOI:** 10.1038/s41598-022-20102-0

**Published:** 2022-09-21

**Authors:** Vivek Kempraj, Soo Jean Park, Donald N. S. Cameron, Phillip W. Taylor

**Affiliations:** Applied BioSciences, Macquarie University, Sydney, NSW Australia

**Keywords:** Behavioural ecology, Coevolution

## Abstract

Humans have used weaver ants, *Oecophylla smaragdina,* as biological control agents to control insect pests in orchards for many centuries. Over recent decades, the effectiveness of weaver ants as biological control agents has been attributed in part to deterrent and oviposition inhibiting effects of kairomones produced by the ants, but the chemical identity of these kairomones has remained unknown. We have identified the kairomone responsible for deterrence and oviposition inhibition by *O. smaragdina*, providing a significant advance in understanding the chemical basis of their predator/prey interactions. Olfactometer assays with extracts from weaver ants demonstrated headspace volatiles to be highly repellent to Queensland fruit fly, *Bactrocera tryoni*. Using electrophysiology and bioassays, we demonstrate that this repellence is induced by a single compound, 1-octanol. Of 16 compounds identified in *O. smaragdina* headspace, only 1-octanol evoked an electrophysiological response from *B. tryoni* antennae. Flies had greatly reduced oviposition and spent significantly less time in an olfactometer arm in the presence of 1-octanol or a synthetic blend of headspace volatiles containing 1-octanol than in the presence of a synthetic blend of headspace volatiles without 1-octanol, or clean air. Taken together, our results demonstrate that 1-octanol is the functional kairomone component of *O. smaragdina* headspace that explains repellence and oviposition deterrence, and is hence an important contributor to the effectiveness of these ants as biological control agents.

## Introduction

Weaver ants are voracious predators and have been used as biological control agents for many centuries to control insect pests in Asia (*Oecophylla smaragdina*) and in Africa (*Oecophylla longinoda*). A common practice in Asia involves establishing a nest on one tree and then connecting it to adjacent trees with bamboo poles, thus enabling the movement of ants throughout the orchard to forage^1,2^. Records of this practice can be found in the 1726 Imperial Encyclopedia of the Ching dynasty and in a regional botany work written by Ji Han in 304^2^. Weaver ants have been found to be effective in controlling insect pests of mango^3–8^, cashew^9,10^, citrus^11,12^, coconut^13^ and cocoa^14,15^. While direct effects of predation by weaver ants are certainly important, recent studies have highlighted that repellence and oviposition deterrence induced by chemical emissions (kairomones) from the ants are also important elements of crop protection conferred by weaver ants^5,16^. Unidentified weaver ant-produced kairomones have been found to inhibit oviposition by fruit fly pests including *Bactrocera jarvisi*^5^, *B. dorsalis* and *Ceratitis cosyra*^17,18^. In the field, damage to mango fruits by *B. jarvisi* is decreased in the presence of *O. smaragdina*^5^. When presented with *O. longinoda*-exposed and unexposed mango fruits in the absence of ants, *B. dorsalis* and *C. cosyra* land less often on ant-exposed fruits and if they do land tend to depart quickly and fail to oviposit^17^. Volatile olfactory cues from *O. smaragdina* induce increases in motility (velocity, active time and distance moved) and reductions in foraging, oviposition and mating propensity in the Queensland fruit fly *Bactrocera tryoni*^19^*.*

While numerous studies have demonstrated fruit fly responses to kairomones from weaver ants, and have strongly implicated such kairomones as an important element of biological control^5,17,18^, the specific compounds responsible remain unknown. Weaver ants are known to emit and deposit a diversity of compounds, including hydrocarbons, esters, fatty acids, terpenes, and alcohols. Hydrocarbons make up ~ 90% of the compounds emitted by *O. smaragdina* with n-undecane being a highly emitted compound (~ 45%)^20^. Identifying kairomones that mediate responses of prey to predators can provide valuable insights to subtle aspects of predator–prey interactions and can also provide insights into how kairomones affect food webs^21,22^. Furthermore, when a predator-released kairomone repels or deters oviposition in a pest species, it can have a significant impact on pest populations^23,24^ and may even be developed as an effective pest management tool. In the present study, we identify a single compound from the headspace volatiles emitted by *O. smaragdina* that is detected by antennae, induces repellence, and deters oviposition in *B. tryoni*. This knowledge significantly advances understanding of predator–prey interactions between weaver ants and fruit flies, and lays the foundations for the development of biologically inspired repellents that could offer a new tool for non-insecticidal, safe, management of economically important fruit flies.

## Results

**Figure 1:**
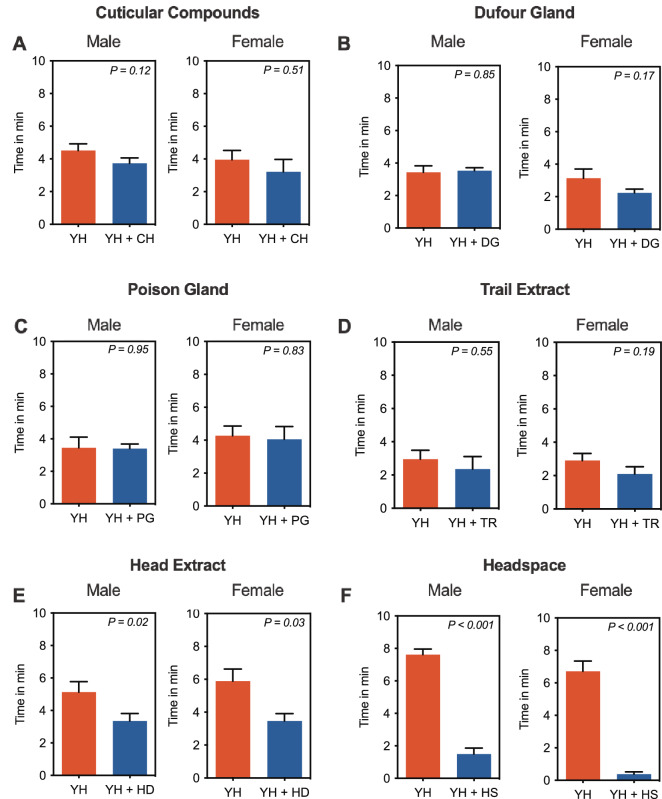
Behavioural response of male and female *B. tryoni* to *O. smaragdina* body extracts and volatiles. (**A**) cuticular compounds; CH, (**B**) Dufour gland; DG, (**C**) poison gland; PG, (**D**) trail extract; TR (**E**) head extract; HD and (**F**) headspace volatiles; HS were tested. Only head extract and headspace volatiles significantly repelled flies. Male and female flies spent significantly more time in control arm (Yeast Hydrolysate; YH) than the treatment arm (Yeast Hydrolysate + Head extract or Yeast Hydrolysate + Headspace voltiles). Error bar represent s.e.m. Significant difference was analysed by paired *t*-test (see Results).

Olfactometer screening of extracts and volatile emissions of *O. smaragdina* revealed that only head extract and headspace volatiles repel *B. tryoni* (Fig. 1). Male and female flies spent significantly more time in the control arm of olfactometers (Male: 5.129 ± 0.65 min (mean ± s.e.m), *t* = 2.383, *df* = 19, *P* = 0.02; Female: 5.885 ± 0.73 min, *t* = 2.249, *df* = 19, *P* = 0.03; Fig. 1e) than in the treatment arm containing head extract (Male: 2.916 ± 0.45 min; Female: 3.459 ± 0.54 min; Fig. 1e). A similar, but a stronger repellence was observed in response to headspace volatiles of *O. smaragdina*. Male and female flies spent significantly more time in the control arm (Male: 7.620 ± 0.34 min; *t* = 9.839; *df* = 19; *P* < 0.001; Female: 6.713 ± 0.73 min, *t* = 9.370, *df* = 19, *P* < 0.001; Fig. 1f) than in the treatment arm containing headspace volatiles (Male: 1.498 ± 0.36 min; Female: 0.3755 ± 0.14 min; Fig. 1f).

**Figure 2:**
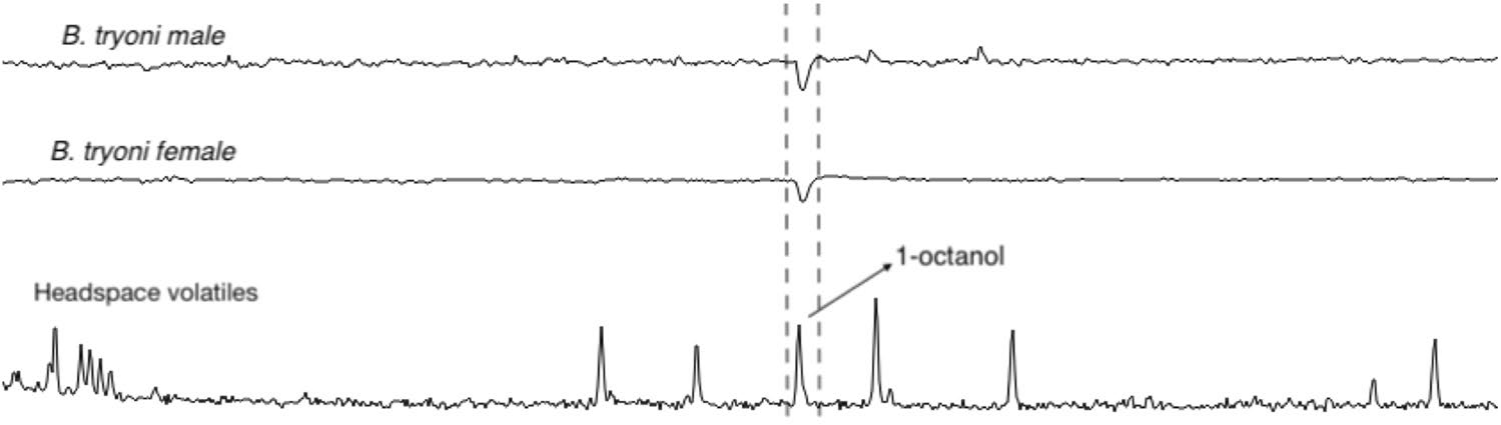
Representative GC-EAD recording of male and female *B. tryoni* response to headspace volatiles of *O. smaragdina*. In both male and female flies the FID peak marked as ‘1-octanol’ was the only compound that elicited consistent response.

**Figure 3:**
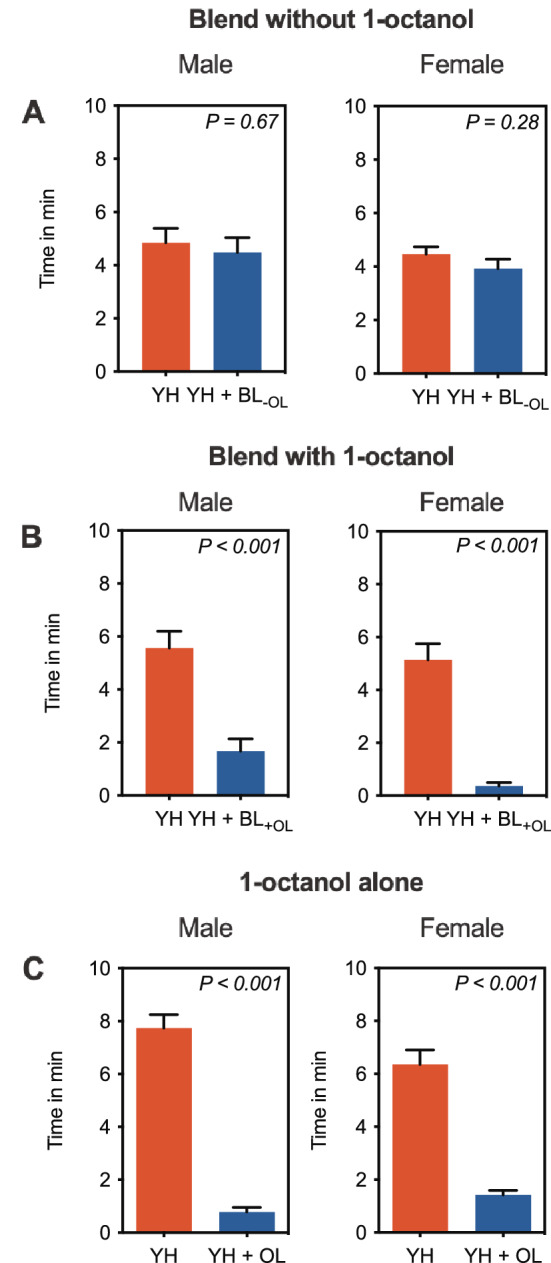
Behavioural response of male and female *B. tryoni* to synthetic blends with (BL_+OL_) or without (BL_−OL_) 1-octanol, and 1-octanol alone (OL). In behavioral assays using synthetic blend of headspace voltiles without 1- octanol, male and female flies spent similar time in control (YH; Yeast hydrolysate) and treatment (YH + BL_−OL_) arms. However, in behavioural assays using a synthetic blend of headspace volatiles with 1-octanol or 1-octanol alone, male and female flies spent significantly (*P* < 0.001) more time in control (YH) than treatment (YH + BL_+OL_) arms. Error bar represent s.e.m. Significant difference was analysed by paired *t*-test (see *Results*).

Headspace volatiles were explored further by Gas Chromatography-Electroantennographic Detection (GC-EAD) to identify compounds that might be responsible for repellence of *B. tryoni*. Both male and female flies responded very consistently to a single compound in the headspace volatiles, and from Gas chromatograph mass spectrometry (GC–MS) analysis the electrophysiologically active compound was found to be 1-octanol (Fig. 2). Although we observed a single compound to be electrophysiologically active on antennae, which are thought to mediate long range olfactory responses, *B. tryoni* do have olfactory receptors on other body parts (e.g., maxillary palps)^25^ and so the possibility remained that other compounds may be detected by organs other than those on antennae and be responsible for repellence. To confirm the functional effect of 1-octanol as a repellent, we prepared two synthetic blends of headspace volatiles, one that contained all components including 1-octanol (BL_+OL_) and one that contained all components except 1-octanol (BL_−OL_). In olfactometer assays, male and female flies were not repelled by the blend BL_−OL_, spending similar amounts of time in the control arm (Male: 4.848 ± 0.53 min; *t* = 0.4238; *df* = 19; *P* = 0.67; Female: 4.459 ± 0.28 min, *t* = 1.106, *df* = 19, *P* = 0.28; Fig. 3a) and the treatment arm (Male: 4.481 ± 0.55 min; Female: 3.922 ± 0.35 min). However, when presented with the blend BL_+OL_, both male and female flies spent significantly more time in the control arm (Male: 5.562 ± 0.64 min; *t* = 4.406; *df* = 19; *P* < 0.001; Female: 5.136 ± 0.61 min, *t* = 7.635, *df* = 19, *P* < 0.001; Fig. 3b) than in the treatment arm (Male: 1.669 ± 0.47 min; Female: 0.366 ± 0.12 min). Next, 1-octanol alone was evaluated for its deterrence of *B. tryoni* to test whether flies responded to 1-octanol outside the context of ant volatiles. Male and female flies spent significantly more time in the control arm (Male: 7.738 ± 0.59 min; *t* = 12.110, *df* = 19, *P* < 0.001; Female: 6.354 ± 0.55 min; *t* = 8.299, *df* = 19, *P* < 0.001; Fig. 3c) than in the treatment arm containing 1-octanol (Male: 0.7765 ± 0.18 min; Female: 1.424 ± 0.17 min).

**Figure 4:**
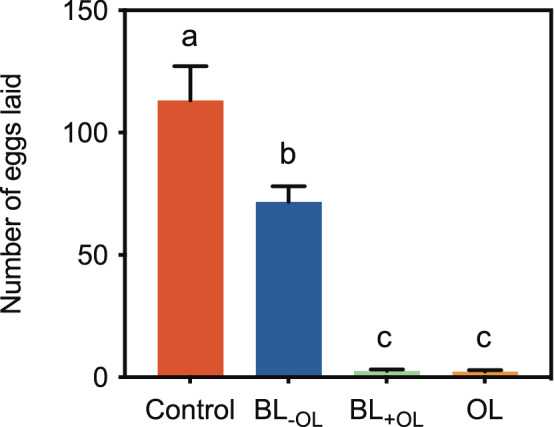
1-octanol inhibited oviposition by gravid *B. tryoni* females. Gravid females were presented with agarose plates containing oviposition stimulant (OS) alone (control), agarose plates containing OS + synthetic blend of headspace volatiles excluding 1-octanol (BL_−OL_), agarose plates containing OS + synthetic blend of headspace volatiles including 1-octanol (BL_+OL_) and agarose plates containing OS + 1-octanol (OL). Significantly more eggs were laid into control and BL_−OL_ than into BL_+OL_ and OL. Error bars represent s.e.m. Significant difference is denoted by different letters (ANOVA followed by Tukey’s test; *P* < 0.001; n = 10; see *Results*).

In oviposition assays, gravid females laid significantly more eggs into control agarose plates (113.2 ± 14.05 eggs; mean ± s.e.m) and BL_−OL_ plates (containing all headspace components except 1-octanol) (71.7 ± 6.33 eggs) than into BL_+OL_ plates (containing all headspace components including 1-octanol) (2.5 ± 0.73 eggs) or OL plates (containing only 1-octanol) (2.2 ± 0.59 eggs)(*F*_3, 36_ = 50.2; *P* < 0.001; Fig. 4). The presence of 1-octanol almost completely inhibited oviposition, but it is important also to note that the effect was by contact or short range olfaction because such inhibition did not carry over to other plates in the same cage. Taken together, our results demonstrate that 1-octanol is responsible for kairomonal effects of repellence and oviposition deterrence in *B. tryoni* that are exposed to olfactory cues from *O. smaragdina* weaver ants.

## Discussion

Kairomones released by predators can significantly influence prey species behaviour and life history^26–29^. Despite numerous studies demonstrating kairomonal effects of olfactory cues released by predators^21,28,30^, there are surprisingly few studies providing chemical characterisation of such kairomones^31–33^.

Although olfactory cues produced by weaver ants (*O. smaragdina* in Asia and Australia and *O. longinoda* in Africa) have been known to have a strong repellent effect on fruit flies^3–8,11,12^, the present study is the first to chemically identify and demonstrate effects of kairomonal components. Active compounds in the headspace appear to originate in the head of *O. smaragdina*. 1-octanol was found to be the only one of 16 compounds in the headspace to elicit electrophysiological responses in *B. tryoni* antennae, and the kairomonal function of 1-octanol as a repellent and oviposition deterrent was demonstrated using bioassays that presented headspace blends with and without 1-octanol. GC–MS analysis of the body extracts and volatile emissions revealed 1-octanol in the head extract and headspace volatiles. 1-Octanol was previously reported from head extracts and mandibular glands of *O. smaragdina*^20,34,35^, and also in headspace^20^. Previous studies have found that 1-octanol in honeybee alarm pheromone repels the parasitic mite, *Varroa jacobsoni*^36^. Octanol (unspecified configuration) has been reported as a minor component in the endocrine secretions of cockroaches, although no function has been identified^37^. 1-Octanol in essential oils of plants has been reported as a biting deterrent in the mosquito *Aedes aegypti*^38^ and as an oviposition deterrent in the Asian corn borer, *Ostrinia furnacalis*^39^. A recent study has shown 1-octanol to be a major component of the alarm pheromones in a mammal, the Bank vole *Myodes glareolus*^40^. However, the function of 1-octanol in *O. smaragdina* is currently unknown. Given the alarm pheromone function of 1-octanol in honeybees, also a social hymenopteran, a similar function warrants investigation in *O. smaragdina*.

Gravid *B. tryoni* rely on fruit volatiles when detecting and choosing fruits for oviposition^41–43^. We used γ-octalactone, a strong oviposition stimulant of *B. tryoni*^44^ and a short-range attractant in some tephritid fruit flies^45^, to establish a high baseline of oviposition in order to demonstrate the substantial inhibitory effects of 1-octanol. In oviposition assays, 1-octanol in the presence or absence of other *O. smaragdina* headspace components over-rode the oviposition-stimulating effect of γ-octalactone resulting in very low levels of oviposition.

Identifying and characterizing predator-released kairomones paves the way for more detailed studies of how prey behaviour and food web structure can be affected by such public information. Identifying the predator-released kairomones that influence oviposition by *B. tryoni* and other fruit flies also provides foundations for the development of new, sustainable, pest management tools. Kairomone-based repellents and oviposition deterrents, such as 1-octanol, could potentially be exploited to protect crops and reduce reliance on environmentally harmful insecticides. In addition to effects on gravid female *B. tryoni*, 1-octanol was found to be repellent to male flies and could potentially contribute to reduced mating in pest populations.

## Materials and methods

### Insects

*Bactrocera tryoni* were obtained from a colony that originated from infested fruit collected in central coastal New South Wales and had been maintained in a controlled environment laboratory (25 ± 0.5 °C, 65 ± 5% RH, photoperiod of 11.5:0.5:11.5:0.5 light: dusk: dark: dawn) at Macquarie University for 32 generations. From emergence, adult flies were fed yeast hydrolysate, sugar and water ad libitum and were used in experiments when 10 to 15 days old, when sexually mature^46^. Major workers of *O. smaragdina* were collected from five different colonies in the vicinity of Mareeba Research Facility, Department of Agriculture and Fisheries, QLD, Australia (17.00724°S, 145.42984°E).

### Chemicals

Authentic standards of 1-hexanol, decane, *p*-cymene, D-limonene, γ-terpinene, 1-octanol, dihydromyrcenol, undecane, nonanal, dodecane, tridecane, 1-tetradecene, tetradecane, pentadecane, hexadecane, heptadecane (all known components of emissions produced by *O. smaragdina*)^20^ and hexane were purchased from Sigma-Aldrich. All chemicals were of analytical grade (≥ 98% purity) and were used without further purification.

### Collection of body & gland extracts, volatile emissions, and trail extracts

Cuticular compounds, head extracts, gland extracts (Dufour and poison glands), headspace volatiles and trail extracts of *O. smaragdina* were collected as described by Kempraj et al.^20^. For cuticular compounds, individual ants (n = 100) were dipped in 10 mL of hexane for 10 s. For head extracts, heads of ants (n = 10) were removed with dissection scissors and immediately placed in 1.5 mL of hexane in a glass vial for 24 h. The extraction time for cuticular compounds and head extract was crucial in achieving differentiation in the compounds extracted. The extended extraction time for head extracts enabled extraction of glandular compounds present in the head (mandibular glands, intramandibular glands, propharyngeal and postpharyngeal glands), whereas the short extraction time for cuticular compounds was enough to extract compounds on the cuticle without significant extractions from glands. For gland extracts, Dufour and poison glands were dissected from the abdomen and remnant tissues were carefully removed using fine forceps. Clean glands (n = 10) were immediately placed into 1.5 mL of hexane in a glass vial. Glands were extracted by standing the vial at room temperature for 24 h. Headspace volatiles present in the air surrounding the ants was collected using an air entrainment system. Ten ants were placed in a cylindrical glass chamber (120 mL capacity) with an inlet and outlet and were allowed to acclimatize for 30 min prior to collection of volatiles. A charcoal filter was connected to the inlet (4 mm ID) of the glass chamber using Tygon tubing (E-3603). The outlet of the glass chamber was connected to a Tenax tube (50 mg, Scientific Instrument Services Inc, Tenax-GR Mesh 60/80, packed in 6 × 50 mm glass tubes) fitted to a screw cap with O-ring. Nine chambers containing ants and one empty control chamber were set up for each run. Headspace volatiles were adsorbed onto Tenax at a flow rate of 0.5 L/min for 30 min by pulling air from the outlet using a pump (KNF Pumps, Model no. NMP850.1.2KNDCB, Switzerland). For trail extracts, we found a metal fence that served as a regular path to transport food and other materials to the nest by *O. smaragdina*. Prior to collection, the section of metal fence (ca.3 m) that the ants used to commute was rinsed with acetone (100 mL) to remove pre-existing trail chemicals. The ants were allowed to make a trail on the rinsed section of the mesh for 24 h. Between 2 and 4 pm Standard Australian Time (when weaver ants are highly active) the metal wire was rinsed, section by section, with a total of 100 mL hexane into a 500 mL glass beaker. The trail wash was concentrated under a gentle stream of clean air down to approximately 10 mL. All collections were at least ten replicates and stored at 4 °C until further processing. Samples of body extracts and gland extracts were treated with a drying agent (sodium sulfate) and by gravity filtration with a glass wool plugged Pasteur pipette to remove water and debris. Samples free from water and debris were concentrated under a gentle stream of nitrogen gas. Cuticular compound samples were concentrated to 1 mL while Dufour’s gland, poison gland and head samples were concentrated to 0.5 mL. Trail samples were filtered to remove solid matter and concentrated to 1 mL under a gentle stream of nitrogen gas. Headspace volatile samples did not require further processing. All processed samples were stored at − 20 °C until analysis.

### Gas chromatography mass spectrometry (GC–MS) analysis

GC–MS analysis of all samples were carried out on a Shimadzu GC–MS TQ8030 spectrometer equipped with a split/splitless injector and SH RTX-5MS (30 m × 0.25 mm, 0.25 µm film) fused silica capillary column. Carrier gas was helium (99.999%) at a flow rate of 1 mL/min. An aliquot of 1 µL was injected in splitless mode, with injector temperature set at 270 °C. The temperature program was as follows: 50 °C for 1 min, increased to 280 °C at 10 °C min^−1^ and increased to 300 °C at 5 °C min^−1^. The ion source and transfer line temperatures were 200 °C and 290 °C respectively. The ionization method was electron impact at a voltage of 70 eV. Spectra were obtained over a mass range of m/z 45–650. For the identification of compounds, mass fragmentation patterns were compared with NIST library (NIST17-1, NIST17-2, NIST17s) and Kovats retention indices were compared with literature values. The identities of the compounds were confirmed by comparing retention index and fragmentation patterns of each compound with authentic standards.

### Electrophysiology

Coupled Gas Chromatography-Electroantennographic Detection (GC-EAD) recordings were made using Ag-glass microelectrodes filled with electroconductive gel (Spectra 360, Parker Laboratories Inc., USA) (n = 6). A male or gravid female of *B. tryoni* was subdued by chilling, and the head was separated from the body using a microscalpel. The base of the head was then fixed to the tip of the gel-filled indifferent electrode. The tip of an antenna was placed in contact with the recording electrode and was slightly inserted into the gel to stabilize the antenna. The signals were passed through a high impedance amplifier (UN-06, Syntech, Hilversum, The Netherlands). Headspace samples were tested by injecting of 1 µl of sample into the GC column. Effluent from the GC column was simultaneously directed to the antennal preparation and the GC detector at a split ratio of 1.5:1, respectively. Separation of compounds was achieved on a Agilent GC 7890B equipped with a split/splitless injector and a flame ionization detector (FID), using an HP-5 column (30 m, 0.32 mm ID, 0.25 μm film, Agilent, CA, US). The carrier gas was hydrogen (99.999%) (BOC, North Ryde, NSW, Australia) at a flow rate of 3.0 mL/min. The injector temperature was 270 °C. The oven temperature was maintained at 45 °C for 2 min, and then increased to 250 °C at 10 °C min^−1^. The outputs from the EAG amplifier and the FID were monitored simultaneously by GcEad software ver. 1.2.5 (Syntech, Kirchzarten, Germany). Peaks eluting from the GC column were judged to be active if they elicited EAD activity in six or more of the ten coupled runs. The identities of FID peaks were confirmed by GC–MS (Shimadzu TQ8030) operating at the same GC conditions with the same type of column (5% diphenyl and 95% dimethyl polysiloxane).

### Preparation of synthetic blends of headspace volatiles

GC–MS results of weaver ant headspace samples guided the preparation of two synthetic blends. The 16 identified headspace compounds^20^ were used to prepare two synthetic blends that matched the relative abundance of compounds in the natural blend. One synthetic blend contained all the headspace components including 1-octanol (BL_+OL_) (BL = Blend; OL = 1-octanol), while the other synthetic blend contained all the headspace components except 1-octanol (BL_−OL_). Stock solutions of the headspace compounds with a concentration range of 5.0–10.0 mg/mL in hexane were prepared in 10 mL volumetric flasks. The stock solutions were run through GC to obtain response factors for the given concentration. The response factor of undecane was used as a reference to adjust the volumes of each compound added to the synthetic blend. The calculated volumes of the compounds were added to a 10 mL volumetric flask. The flask was filled with hexane to the mark and inverted several times to mix the blend well. The synthetic blend was run through GC to confirm if the relative gas chromatographic (GC) intensities of the compounds were consistent with that in the natural headspace volatile extract. Preparing a synthetic blend and comparing GC intensities were repeated several times until the relative GC intensities were consistent with that in the natural headspace volatile extract. The concentration of undecane, the reference compound, was arbitrary each time but in a range of 10.0 to 15.0 μg/mL. The GC conditions used in this process were the same as the above GC–MS analysis, except that 1 μl of sample was injected at split mode (a ratio of 1:60).

### Olfactometer bioassays

An acrylic four-arm olfactometer (120 mm diameter; see Fig. S1) was used to assess behavioural responses of male and female *B.* *tryoni* to extracts of cuticle, Dufour gland, Poison gland, Trail and head and volatile emissions of weaver ants as well as synthetic blends (BL_+OL_, BL_−OL_) or 1-octanol (OL) alone. Prior to each experiment, olfactometers were washed with a non-ionic detergent solution, rinsed with ethanol and distilled water, and left to air dry. Experiments were conducted in a controlled environment room (25 ± 2 °C, 60% RH). To provide traction for the walking insects, filter paper (Whatmann No. 1, 12 cm diameter) was placed on the floor of the central area. The room was illuminated from above by uniform lighting from white LED lights. Individual flies (10–15 days old, without access to food over the preceding 24 h, but with access to water) were introduced to the olfactometer through a hole in the floor. Each fly was given 5 min to acclimatize in the olfactometer, after which the experiment was run for 10 min. The olfactometer was rotated 90° after each replicate to eliminate any directional bias. Air was drawn through the central hole at 200 ml min^−1^ and subsequently exhausted into the room. The central arena of the olfactometer was divided into four discrete odour fields corresponding to each of four inlet arms. A choice test was performed that used two opposite arms and the other two arms were closed and were not used in the test. One arm was for treatment and the opposite arm was control. Test samples (extracts, volatile emissions, BL_+OL_, BL_−OL_ or OL-1.17% v/v (10 μl; the concentration of 1-octanol used was similar to the concentration of 1-octanol present in the natural headspace sample)^20^ and yeast hydrolysate solution (YH; 6% w/v, 10 μl, a feeding stimulant) were tested individually. The test sample was pipetted onto filter paper strips that were placed into the treatment cylinder through which air was drawn to one arm of the olfactometer, while the cylinder through which air was drawn to the control arm of the olfactometer contained only YH (10 μl). Fly activity was video recorded. The time spent in each arm was analysed using BORIS software ver. 7.9.6^47^. Twenty replicates were conducted for each type of sample.

### Oviposition assay

To determine whether 1-octanol is key in deterring oviposition by gravid female flies, oviposition responses of gravid females were assessed using agarose plates containing an oviposition stimulant (OS; γ-octalactone)^44^. Number of eggs oviposited on agarose plates containing synthetic blends of weaver ant headspace volatiles (BL_+OL_, BL_−OL_; 10 µl) or 1-octanol (OL; 1.17% v/v in hexane; 10 µl) was compared with number of eggs oviposited on agarose plates containing OS alone (control). Agarose (0.8 g in 100 ml water) was melted in a microwave oven, and then cooled to ~ 60 °C. OS (0.05% v/v in hexane; 10 µl) alone or in combination with BL_+OL_, BL_−OL_ or OL (10 µl) was added. This mixture was poured into pre-cooled Petri dishes, covered, and stored for 10 min at 4 °C. Agarose plates containing OS alone (control) and OS combined with BL_+OL_, BL_−OL_ and OL were all provided to gravid females at the same time as a multiple-choice test (50 gravid females; 13–15 days old from mixed sex cages) in mesh cages (45 × 45 × 45 cm, BugDorm-4S4545). The plates were placed at four corners of the mesh cage and were separated by ~ 40 cm from each other. After 24 h, eggs laid in each plate were counted under a dissecting microscope (Olympus SZX12, Japan). Ten replicates of the assay were conducted.

### Statistical analysis

Data from olfactometer assays were subjected to paired *t* tests to assess whether the amount of time spent by flies in the olfactometer arms differed significantly between control and treatment. Data from oviposition assays were subjected to one-way ANOVA followed by Tukey’s multiple comparison test to compare the treatments. Statistical analysis was preformed using GraphPad Prism, version 9.0 (GraphPad Software LLC, USA).

## Supplementary Information

Supplementary Information.

## Data Availability

The datasets generated and analysed during this current study are available from ResearchGate (https://doi.org/10.13140/RG.2.2.20780.74882).
