# Supplementary material for: 1-Octanol emitted by Oecophylla smaragdina weaver ants repels and deters oviposition in Queensland fruit fly
Source: Sci Rep. 2022 Sep 21;12:15768. doi: 10.1038/s41598-022-20102-0 (PMC9492660; doi:10.1038/s41598-022-20102-0)
Supplement: Supplementary file 1 — Supplementary Information. [file 41598_2022_20102_MOESM1_ESM.pdf]

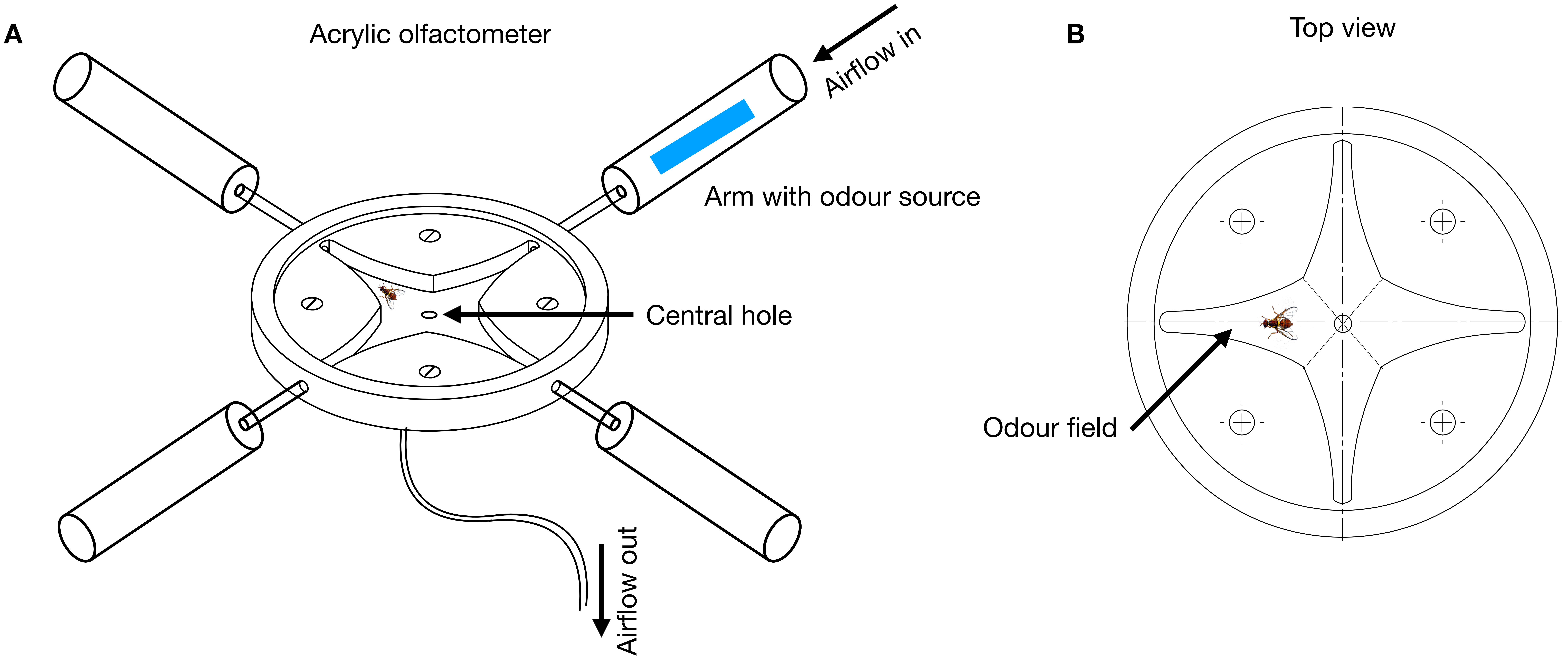

Figure S1. Olfactometer design used in this study. (**A**) Schematic representation of 4-arm olfactometer used in our study. (**B**) The top view of the olfactometer.
